# Diabetes Mellitus and Its Correlates in an Iranian Adult Population

**DOI:** 10.1371/journal.pone.0026725

**Published:** 2011-10-28

**Authors:** Asieh Golozar, Hooman Khademi, Farin Kamangar, Hossein Poutschi, Farhad Islami, Christian C. Abnet, Neal D. Freedman, Philip R. Taylor, Paul Pharoah, Paolo Boffetta, Paul J. Brennan, Sanford M. Dawsey, Reza Malekzadeh, Arash Etemadi

**Affiliations:** 1 Digestive Disease Research Institute, Shariati Hospital, Tehran University of Medical Sciences, Tehran, Iran; 2 Division of Cancer Epidemiology and Genetics, National Cancer Institute, Bethesda, Maryland, United States of America; 3 Department of Public Health Analysis, School of Community Health and Policy, Morgan State University, Baltimore, Maryland, United States of America; 4 International Agency for Research on Cancer, Lyon, France; 5 Cancer Research UK, Department of Oncology, Cambridge University, Cambridge, United Kingdom; 6 The Tisch Cancer Institute, Mount Sinai School of Medicine and Institute for Translational Epidemiology, New York, New York, United States of America; 7 International Prevention Research Institute, Lyon, France; Universidad Peruana Cayetano Heredia, Peru

## Abstract

The rising epidemic of diabetes imposes a substantial economic burden on the Middle East. Using baseline data from a population based cohort study, we aimed to identify the correlates of diabetes mellitus (DM) in a mainly rural population from Iran. Between 2004 and 2007, 50044 adults between 30 and 87 years old from Golestan Province located in Northeast Iran were enrolled in the Golestan Cohort Study. Demographic and health-related information was collected using questionnaires. Individuals' body sizes at ages 15 and 30 were assessed by validated pictograms ranging from 1 (very lean) to 7 in men and 9 in women. DM diagnosis was based on the self-report of a physician's diagnosis. The accuracy of self-reported DM was evaluated in a subcohort of 3811 individuals using fasting plasma glucose level and medical records. Poisson regression with robust variance estimator was used to estimate prevalence ratios (PR's). The prevalence of self-reported DM standardized to the national and world population was 5.7% and 6.2%, respectively. Self-reported DM had 61.5% sensitivity and 97.6% specificity. Socioeconomic status was inversely associated with DM prevalence. Green tea and opium consumption increased the prevalence of DM. Obesity at all ages and extreme leanness in childhood increased diabetes prevalence. Being obese throughout life doubled DM prevalence in women (PR: 2.1; 95% CI: 1.8, 2.4). These findings emphasize the importance of improving DM awareness, improving general living conditions, and early lifestyle modifications in diabetes prevention.

## Introduction

Chronic diseases, including diabetes mellitus (DM), have replaced infectious diseases as the main causes of morbidity and mortality in the developing world [1], [2]. Seventy percent of diabetics in 2010 lived in low- and middle-income countries, and the greatest relative increase in the burden of DM is expected to occur in Africa and the Middle East, an approaching epidemic warranting further study [3]–[5].

In Iran, the prevalence of DM adjusted for the world population was predicted to reach 8% in 2010 [5], and the total health expenditure for DM in 2010 was estimated to be approximately 600 million US dollars [6]. As in other parts of the world, obesity has been the most consistent risk factor for DM in studies conducted in Iran [7], [8]. Most of these studies, however, have been conducted in large metropolitan areas. Substantial differences between urban and rural populations exist in Iran, particularly in terms of ethnicity, socio-economic status (SES), and habits. As such, previous observations in urban populations may not apply to rural areas.

Golestan Province is a largely rural province located in northeastern Iran. The prevalence of obesity in Golestan is higher than in most other parts of Iran and many high-income countries [9]. Golestan also lags behind some other parts of Iran in terms of its economic and lifestyle development, so studies in Golestan provide an opportunity to assess disease etiologies in a population in the early stages of economic transition.

Golestan Province has attracted scientific attention mostly because of its very high rates of esophageal cancer [10]. Between 2004 and 2007, 50044 Golestan adults (including almost 40000 rural residents) were enrolled in the Golestan Cohort Study (GCS), which was primarily designed to investigate risk factors for esophageal cancer. Baseline data from this cohort, gave us the opportunity to perform a cross-sectional evaluation of obesity and other less-studied risk factors for diabetes in this mainly rural population.

## Materials and Methods

The design of the GCS has been described before [11]. Briefly, the GCS is a prospective population-based cohort study, launched in January 2004, which has recruited 50044 adults between 30 and 87 years old from Golestan Province.

Using systematic clustering based on household numbers, a total of 39399 individuals from 326 rural villages and 10645 urban residents were enrolled. Demographics and baseline information including age, sex, education, ethnicity, place of residence, number of owned household appliances, and history of tobacco and opium use were collected using a structured lifestyle questionnaire. Anthropometric data were measured and samples of blood, urine, hair and nails were gathered from the participants by a trained technician after the interview.

Education (highest level attained) and appliance ownership, including bath in the residence, personal car, motorbike, black and white TV, color TV, refrigerator, freezer, vacuum cleaner and washing machine, were used as indicators of SES. Using multiple correspondence analysis, we created a wealth score based on the appliance ownership variables. These scores were calculated and participants were categorized into wealth score quartiles [12].

Given the lifestyle of this mainly rural population, most of the activities individuals have are at work. As a result, only physical activity at work was looked at in this analysis. Two questions were asked about individuals' work activity: if the person worked every month throughout the year, and if intense physical activity was part of the daily work. Three levels of occupational physical activity were defined based on the answers to these questions: intense physical activity at work, non-intense but regular physical activity and non-intense irregular physical activity.

Individuals were considered tobacco users if they had smoked cigarettes or had used nass, hookah or a pipe at least once a week for a period of 6 months or more. Individuals were categorized into these groups: never smokers, former cigarette smokers, current cigarette smokers, and those who smoked other forms of tobacco (nass, hookah, or a pipe). Current cigarette smokers were further divided into light and heavy smokers if they fell below or above the median pack years for the nondiabetic smokers. Likewise, opium users were defined as those who consumed opium at least once a week for 6 months or more. The self-reported use of opium is a reliable and valid indicator of opium exposure in this population [13].

Systolic and diastolic blood pressures were measured twice in each arm in the sitting position and averaged. Participants were considered as being hypertensive if they either reported a physician's diagnosis of hypertension, were using anti-hypertensive medication, or fulfilled the criteria of the Joint National Committee on Prevention, Detection, Evaluation, and Treatment of High Blood Pressure (JNC-7) (average systolic blood pressure above ≥140 mmHg, or average diastolic blood pressure above ≥90 mmHg) [14]. DM was self-reported based on this question: “Have you ever been diagnosed by a doctor as having diabetes mellitus?”.

Green tea consumption was categorized based on both the frequency and amount of drinking. Non-drinkers did not drink green tea at all, occasional green tea drinkers drank it less than once a week, and frequent drinkers drank it at least once a week, but not every day. Those who drank green tea every day were divided into low and high intake groups based on whether they drank less or more than the median (600 ml), respectively. Black tea consumption was divided into quartiles based on average daily drinking, since there were very few people who didn't drink black tea every day.

Oral health status was summarized using the sum of the number of decayed, missing, or filled teeth (DMFT), and categorized into 3 levels: <20, 21–31, and 32.

Body mass index (BMI), as a measure of overall obesity, was calculated by dividing measured weight (kg) by the square of the measured height (m), and categorized using the World Health Organization (WHO) cutoffs: underweight (BMI<18.5 kg/m^2^), normal (18.5≤BMI<25 kg/m^2^), overweight (25≤BMI<30 kg/m^2^), and obese (BMI≥30 kg/m^2^) [15]. Waist circumference (WC) was used as a measure of abdominal obesity. Individuals were categorized as either normal or high risk (WC>102 cm in men and >88 cm in women) according to the adult treatment panel (ATP) III criteria [16]. Additionally, participants were also categorized into quintiles of WC.

**Figure 1:**
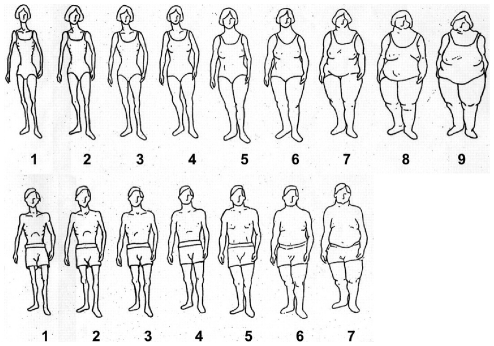
Body size pictograms used in the Golestan Cohort Study.

Individuals' body size perceptions at ages 15 and 30 were assessed using a set of drawings (pictograms), ranging from very lean to obese. These pictograms were developed by Stunkard et al [17], and have been shown to have good accuracy for anthropometric assessment in this population [18]. The pictograms were scored from 1 to 7 in men, and from 1 to 9 in women (Figure 1). The highest two categories of pictogram score were combined together due to the relatively small number of observations in these categories. Obesity was defined as a pictogram score of 5 and above [17], Change in pictogram score between ages 15 and 30 was used to assess the association between change in body size during adulthood and DM. Study participants were categorized into four categories: no change, decrease, slight increase (a 1 or 2 category increase) and prominent increase (a more than 2 category increase).

Five years after recruitment, fasting plasma glucose (FPG) level was measured for a random sample of 3811 cohort participants. The same baseline questionnaire (including DM self-report) was again administered at the time of blood draw. Individuals who had FPG≥126 mg/dl (7.0 mmol/l) (the recommended cutoff of the American Diabetes Association [19]) or were under anti-diabetic treatment were categorized as having confirmed DM. This information was used to assess the sensitivity and specificity of self-reported DM in this study.

The GCS was approved by the Institutional Review Boards of the Digestive Disease Research Center of Tehran University of Medical Sciences, the US National Cancer Institute (NCI), and the World Health Organization International Agency for Research on Cancer (IARC). All participants gave written informed consent before enrollment.

### Statistical analysis

The World Standard Population 2000–2005 developed by the WHO [20] and the 2009 population provided by the Statistical Center of Iran [21] were used for world and national age-standardizations, respectively, using the direct age-standardization method.

We used Poisson regression with robust variance estimator to get unbiased estimates of prevalence ratios (PR). Poisson regressions with robust variance estimator are useful alternatives to log-binomial models; they work equally well when the model is correctly specified, and are not subject to the convergence difficulties [22].

Multicollinearity was assessed using the variance inflation factor (VIF). Aside from obesity-related covariates, no evidence for serious multicollinearity was observed (all VIFs were below 1.5). Univariate models were first fitted to assess the independent association between each covariate and DM. Potential confounders and mediators were identified using a Directed Acyclic Graph (DAG). Variables included in the DAG were those that have been consistently reported to be associated with DM, have relevant biological mechanisms in the disease process, or have been hypothesized to be associated with DM.

Multivariate models were fitted to assess the direct association between each of the covariates of interest and diabetes. For age, the PR was calculated per 10 years increase in age. Since BMI, WC and pictograms at 15 and 30 years are all measures of obesity, and also showed high VIF (>2.5), separate models were built for each to avoid collinearity. Obesity-related covariates were assessed separately in men and women, but as the effect of BMI on DM was similar in both sexes, only the pooled effect was reported. All these models were further adjusted (according to DAG) for age, ethnicity, place of residence, education and wealth score (2 different indicators of SES), physical activity, tobacco use, opium use, hypertension, green tea consumption, black tea consumption, and DMFT score. Another model was built to assess whether change in body size in early adulthood (between 15 and 30) was associated with DM risk. Since size at a young age is invariably correlated with size later in life, we additionally adjusted this model for body size at 15 years. Finally, the cumulative effect of obesity since 15 years of age was assessed using the combination of obesity at 15 and 30 (pictogram score ≥5) and at the time of recruitment (BMI≥30). Individuals were categorized into 5 groups; never obese, obese at age 15, obese at both ages 15 and 30, obese at age 30 and recruitment, and always obese.

Sensitivity and specificity of self-reported DM were calculated using the data collected 5 years after recruitment. Confirmed DM (defined above) was used as a gold standard for this calculation.

All statistical analyses were performed using STATA statistical software version 11 (Stata Corporation, College Station, TX, USA). We used hotdeck method to impute the missing values for variables with more than 50 missing observations. All tests of hypothesis were conducted at a confidence level of 0.95 under the two-sided alternative.

## Results

The mean age of the cohort participants was 52.1, ranging from 30 to 87 years. 57.6% were female, 74.4% were from Turkmen ethnicity, 78.7% were rural residents and 70.2% were illiterate.

Of the 50044 individuals recruited into the cohort, 3453 reported having DM at baseline, a crude prevalence of 6.9% (95% CI: 6.7%, 7.1%). The prevalence standardized to the national and worldwide population was 5.7% and 6.2%, respectively. Stratified by ethnicity, the prevalence was 5.9% (95% CI: 5.7%, 6.2%) in Turkmens and 9.7% (95% CI: 9.2%, 10.3%) in non-Turkmens.

In the subcohort of 3811 participants with available FPG measurements, the crude prevalence of confirmed DM was 10.9% (95% CI: 9.9, 11.9) and the prevalence after standardization to the national and world population was 9.8% and 10.2%, respectively. The sensitivity and specificity of self-reported DM in this subcohort were 61.5% and 97.6%, respectively.

Crude and adjusted PR estimates for DM are reported in Tables 1–4. The adjusted prevalence of diabetes increased 21% for every 10-year increase in age. The adjusted PRs of DM associated with non-Turkmen ethnicity and residence in an urban area were 1.6 (95% CI: 1.5, 1.8) and 1.1 (95% CI: 1.0, 1.2), respectively. The prevalence of DM was significantly lower in those with a higher wealth score and higher educational level (*P* value for trend <0.0001).

**Table 1:** Study Subject Demographics by Self-Reported Diabetes in the Golestan Cohort Study.

	Diabetics, N (%)	Non-Diabetics, N (%)	Crude PR	95% CI	Adjusted PR	95% CI^a^
	3453 (6.9)	46586 (93.1)				
**Age, mean (SD)**	54.6 (8.7)	51.9 (9.0)	1.32^b^	1.28, 1.37	1.21^b^	1.16, 1.26
**Gender, N (%)**						
Female	2351 (8.2)	26448 (91.8)	1	—	1	—
Male	1102 (5.2)	20138 (94.8)	0.64	0.59, 0.68	0.98	0.89, 1.08
**Ethnicity, N (%)**						
Turkmen	2209 (5.9)	35039 (94.1)	1	—	1	—
Non-Turkmen	1244 (9.7)	11547 (90.3)	1.64	1.53, 1.78	1.59	1.51, 1.82
**Residence, N (%)**						
Rural	2451 (6.2)	36945 (93.8)	1	—	1	—
Urban	1002 (9.4)	9641 (90.6)	1.51	1.41, 1.62	1.08	0.99, 1.18
**Wealth score, N (%)**						
Low	1180 (9.1)	11829 (90.9)	1	—	1	—
Low-Medium	896 (8.3)	11452 (92.7)	0.80	0.74, 0.87	0.88	0.81, 0.96
Medium-High	660 (6.5)	9435 (93.5)	0.72	0.66, 0.79	0.78	0.71, 0.86
High	717 (4.9)	13868 (95.1)	0.54	0.50, 0.59^c^	0.63	0.57, 0.70^c^
**Education, N (%)**						
Illiterate	2573 (7.3)	32540 (92.7)	1	—	1	—
Up to High School	822 (5.9)	13039 (94.1)	0.81	0.75, 0.87	0.92	0.84, 1.01
Higher Education	58 (5.5)	1006 (94.5)	0.74	0.58, 0.96^c^	0.85	0.65, 1.11^c^

PR: prevalence ratio, CI: confidence interval, SD: standard deviation.

a Variables included in the model: age, sex, place of residence, race, education, wealth score, physical activity, hypertension, opium, tobacco smoking, green and black tea consumption, DMFT score, and BMI.

b PR (95% CI) for every 10 year increase in age.

c P-value for trend <0.0001.

**Table 2 pone-0026725-t002:** Subject Characteristics by Self-Reported Diabetes in the Golestan Cohort Study.

	Diabetics, N (%)	Non-Diabetics, N (%)	Crude PR	95% CI	Adjusted PR	95% CI^a^
	3453 (6.9)	46586 (93.1)				
**Tobacco use, N (%)**						
Never-smoker	2934 (7.5)	36259 (92.5)	1	—	1	—
Former smoker	215 (6.7)	2986 (93.3)	0.90	0.78, 1.02	1.00	0.86, 1.17
Current light smoker	75 (3.3)	2175 (96.7)	0.44	0.36, 0.56	0.70	0.56, 0.88
Current heavy smoker	118 (3.7)	3053 (96.3)	0.50	0.42, 0.60	0.77	0.64, 0.94
Ever-hookah, nass or pipe user	107 (4.9)	2070 (95.1)	0.66	0.55, 0.79^b^	0.68	0.56, 0.82^b^
**Opium use, N (%)**						
Never-user	2908 (7.0)	38633 (93.0)	1	—	1	—
Ever-user	545 (6.4)	7953 (93.6)	0.92	0.84, 1.00	1.38	1.25, 1.52
**Hypertension, N (%)**						
Normotensive	1210 (4.2)	27417 (95.8)	1	—	1	—
Hypertensive	2230 (10.5)	18967 (89.5)	2.48	2.32, 2.65	1.82	1.70, 1.96
**Physical activity at work, N (%)**						
Irregular non-intense	2492 (8.1)	28177 (91.9)	1	—	1	—
Regular non-intense	774 (5.7)	12846 (94.3)	0.70	0.65, 0.76	0.81	0.75, 0.89
Regular or irregular intense	187 (3.3)	5563 (96.7)	0.40	0.35, 0.46^b^	0.58	0.50, 0.68^b^
**Green Tea, N (%)**						
None	2712 (6.7)	38044 (93.3)	1	—	1	—
Less than once a week	275 (8.4)	2985 (91.6)	1.29	1.14, 1.45	1.21	1.08, 1.36
Weekly	174 (7.5)	2151 (92.5)	1.13	0.98, 1.31	1.05	0.91, 1.22
Light daily (<600 ml)	133 (8.9)	1358 (91.1)	1.32	1.12, 1.56	1.25	1.06, 1.47
Heavy daily (≥600 ml)	127 (9.6)	1193 (90.4)	1.44	1.21, 1.70^b^	1.24	1.05, 1.47^b^
**Black Tea, N (%)**						
Q1 (≤690 ml)	1063 (7.8)	12531 (92.2)	1	—	1	—
Q2 (691–1035 ml)	786 (6.8)	10843 (93.2)	0.90	0.83, 0.98	0.96	0.88, 1.04
Q3 (1036–1500 ml)	765 (6.5)	11090 (93.5)	0.84	0.77, 0.92	0.92	0.84, 1.00
Q4 (>1500 ml)	802 (6.7)	11202 (93.3)	0.85	0.78, 0.93^b^	1.02	0.94, 1.12^b^
**DMFT Categories, N (%)**						
<20	1006 (6.1)	15447 (93.9)	1	—	1	—
20–32	1113 (6.7)	15464 (93.3)	1.10	1.01, 1.19	1.04	0.96, 1.14
32	1322 (7.8)	15526 (92.2)	1.28	1.19, 1.39^b^	1.07	0.98, 1.17

PR: prevalence ratio, CI: confidence interval, DMFT: decayed, missing, or filled teeth.

a Variables included in the model: age, sex, place of residence, race, education, wealth score, physical activity, hypertension, opium, tobacco smoking, green and black tea consumption, DMFT score, and BMI.

b P-value for trend <0.0001.

**Table 3 pone-0026725-t003:** Body mass index and Waist Circumference by Self-Reported Diabetes in the Golestan Cohort Study.

	Diabetics, N (%)	Non-Diabetics, N (%)	Crude PR	95% CI	Adjusted PR	95% CI^a^
	3453 (6.9)	46586 (93.1)				
**Body mass index (BMI), N (%)**						
Underweight (BMI<18.5)	43 (1.8)	2294 (98.2)	0.49	0.36, 0.67	0.51	0.38, 0.70
Normal (BMI 18.5–24.9)	668 (3.7)	17188 (96.3)	1	—	1	—
Overweight (BMI 25.0–29.9)	1401 (8.3)	15489 (91.7)	2.22	2.03, 2.43	1.83	1.66, 2.01
Obese (BMI≥30)	1328 (10.4)	11389 (89.6)	2.77	2.53, 3.03^b^	1.95	1.76, 2.16^b^
**Waist circumference, N (%)**						
**Men**						
**ATP III Criteria**						
Normal (<102 cm)	549 (3.7)	14387 (96.3)	1	—	1	—
Risky (≥102 cm)	552 (8.8)	5747 (91.2)	2.38	2.13, 2.67	2.44	2.17, 2.73
**Quintiles**						
Q1 (<93 cm)	147 (3.0)	4793 (97.0)	1	—	1	—
Q2 (93–96 cm)	155 (4.0)	3741 (96.0)	1.34	1.07, 1.67	1.37	1.09, 1.70
Q3 (97–100 cm)	245 (5.7)	4072 (94.3)	1.91	1.56, 2.33	2.01	1.65, 2.46
Q4 (101–105 cm)	288 (6.7)	3978 (93.3)	2.27	1.87, 2.76	2.46	2.02, 2.98
Q5 (>105)	265 (7.0)	3542 (93.0)	2.34	1.92, 2.85^b^	2.61	2.14, 3.19^b^
**Women**						
**ATP III Criteria**						
Normal (<88 cm)	204 (2.6)	7601 (97.4)	1	—	1	—
Risky (≥88 cm)	2147 (10.2)	18841 (89.8)	3.91	3.40, 4.51	3.90	3.39, 4.49
**Quintiles**						
Q1 (<93 cm)	389 (6.0)	6,085 (94.0)	1	—	1	—
Q2 (93–97 cm)	466 (8.4)	5,109 (91.6)	1.39	1.22, 1.58	1.51	1.33, 1.72
Q3 (98–102 cm)	474 (8.1)	5,411 (91.9)	1.34	1.18, 1.53	1.53	1.35, 1.75
Q4 (103–105 cm)	465 (8.7)	4,853 (91.3)	1.46	1.28, 1.66	1.72	1.51, 1.95
Q5 (>108)	556 (10.0)	4,984 (90.0)	1.67	1.47, 1.89^b^	1.98	1.74, 2.24^b^

PR: prevalence ratio, CI: confidence interval.

a All models were adjusted for age, place of residence, race, education, wealth score, physical activity, hypertension, opium, tobacco smoking, green and black tea consumption, DMFT score. Change in pictogram score between 15 and 30 analysis was further adjusted for pictogram score at 15.

b *P* value for trend <0.0001.

**Table 4:** Body size at ages 15 and 30 by Self-Reported Diabetes in the Golestan Cohort Study.

	Diabetics, N (%)	Non-Diabetics, N (%)	Crude PR	95% CI	Adjusted PR	95% CI^a^
	3453 (6.9)	46586 (93.1)				
**Pictogram at age 15, N (%)**						
**Men**						
1 (slimmest)	157 (7.3)	2004 (92.7)	1.40	1.16, 1.69	1.35	1.12, 1.63
2	285 (5.2)	5205 (94.8)	1	—	1	—
3	284 (4.4)	6157 (95.6)	0.85	0.72, 1.00	0.87	0.74, 1.02
4	216 (5.3)	3878 (94.7)	1.02	0.86, 1.21	1.08	0.91, 1.28
5	84 (4.4)	1814 (95.6)	0.85	0.67, 1.08	0.95	0.75, 1.20
more than 6	76 (6.6)	1080 (93.4)	1.27	0.99, 1.62	1.34	1.04, 1.72
**Women**						
1 (slimmest)	765 (9.3)	7502 (90.7)	1.31	1.17, 1.47	1.20	1.07, 1.34
2	407 (7.1)	5369 (92.9)	1	—	1	—
3	266 (6.9)	3573 (93.1)	0.98	0.85, 1.14	1.02	0.88, 1.18
4	191 (7.1)	2489 (92.9)	1.01	0.86, 1.19	1.04	0.88, 1.22
5	221 (8.7)	2321 (91.3)	1.23	1.05, 1.44	1.24	1.06, 1.45
6	162 (7.4)	2029 (92.6)	1.05	0.88, 1.25	1.04	0.87, 1.24
7	103 (8.5)	1114 (91.5)	1.20	0.98, 1.48	1.16	0.94, 1.43
more than 8	236 (10.3)	2051 (89.7)	1.46	1.26, 1.71^b^	1.32	1.13, 1.55^b^
**Pictogram at age 30, N (%)**						
**Men**						
1 (slimmest)	8 (2.7)	291 (97.3)	0.75	0.37,1.52	0.73	0.36, 1.47
2	95 (3.6)	2559 (96.4)	1	—	1	—
3	272 (4.1)	6446 (95.9)	1.13	0.90,1.42	1.08	0.86, 1.35
4	345 (5.0)	6506 (95.0)	1.41	1.13,1.76	1.29	1.03, 1.60
5	245 (7.0)	3234 (93.0)	1.97	1.56,2.48	1.73	1.37, 2.17
more than 6	137 (11.1)	1102 (88.9)	3.09	2.40,3.98^c^	2.61	2.03, 3.35^c^
**Women**						
1 (slimmest)	91 (5.4)	1585 (94.6)	0.84	0.67,1.05	0.81	0.66, 1.01
2	374 (6.5)	5419 (93.5)	1	—	1	—
3	439 (6.8)	6021 (93.2)	1.05	0.92,1.20	1.05	0.92, 1.20
4	371 (7.3)	4707 (92.7)	1.13	0.99,1.30	1.12	0.98, 1.28
5	366 (8.3)	4027 (91.7)	1.29	1.12,1.48	1.25	1.09, 1.44
6	351 (11.7)	2649 (88.3)	1.81	1.58,2.08	1.71	1.49, 1.97
7	189 (13.6)	1203 (86.4)	2.10	1.78,2.48	1.88	1.59, 2.21
more than 8	170 (16.9)	837 (83.1)	2.61	2.21,3.09^c^	2.18	1.84, 2.59^c^
**Change in Pictogram from 15 to 30, N (%)**						
**Men**						
No change	213 (3.4)	6121 (96.6)	1	—	1	—
Decrease	101 (3.3)	2955 (96.7)	0.98	0.78, 1.24	0.84	0.65, 1.08
Increase≤2	470 (5.7)	7833 (94.3)	1.68	1.44, 1.97	1.55	1.31, 1.83
Increase>2	318 (9.0)	3229 (91.0)	2.67	2.25, 3.16^c^	2.31	1.90, 2.81^c^
**Women**						
No change	366 (6.4)	5372 (93.6)	1	—	1	—
Decrease	463 (6.5)	6651 (93.5)	1.02	0.89, 1.17	0.82	0.71, 0.95
Increase≤2	686 (7.8)	8153 (92.2)	1.22	1.08, 1.38	1.26	1.11, 1.43
Increase>2	836 (11.8)	6272 (88.2)	1.84	1.64, 2.07^c^	1.76	1.55, 2.00^c^

PR: prevalence ratio, CI: confidence interval.

a All models were adjusted for age, place of residence, race, education, wealth score, physical activity, hypertension, opium, tobacco smoking, green and black tea consumption, DMFT score. Change in pictogram score between 15 and 30 analysis was further adjusted for pictogram score at 15.

b *P* value for trend <0.01.

c *P* value for trend <0.0001.

The prevalence of diabetes was approximately 30% lower in current smokers than never-smokers (Table 2). Ever-hookah, nass or pipe smoking was also associated with 32% decrease in the prevalence of diabetes compared to never-smokers. Opium use was similar in diabetics and non-diabetics. However, the adjusted PR of DM associated with opium use was 1.4 (95% CI: 1.3, 1.5).

Hypertension was associated with an 82% increase, and regular or intense physical activity at work was associated with a 42% decrease in DM prevalence (Table 2). There was no association between black tea consumption and diabetes, but green tea consumption was associated with increased prevalence of diabetes (*P* value for trend <0.0001).

Both BMI and WC were associated with diabetes in this population (Table 3). The prevalence of DM was 83–95% higher in overweight and obese people, defined by BMI, and DM prevalence also increased with increasing WC in both men and women (*P* values for trend <0.0001). Extreme leanness and obesity during childhood were associated with a significantly increased prevalence of DM in both sexes (table 4). Additionally, there was a significant stepwise increase in DM prevalence with increasing 30 year-old pictogram scores (*P* values for trend <0.0001). Increase in body size from 15 to 30 years was also associated with an increase in the prevalence of DM. Among those with more than a 2-unit pictogram increase, the PR of DM was 2.3 (95% CI: 1.9, 2.8) in men and 1.8 (95% CI: 1.6, 2.0) in women. Decrease in body size between these ages had an inverse association with DM, although the association was statistically significant only in women (PR: 0.8; 95% CI: 0.7, 1.0).

**Figure 2:**
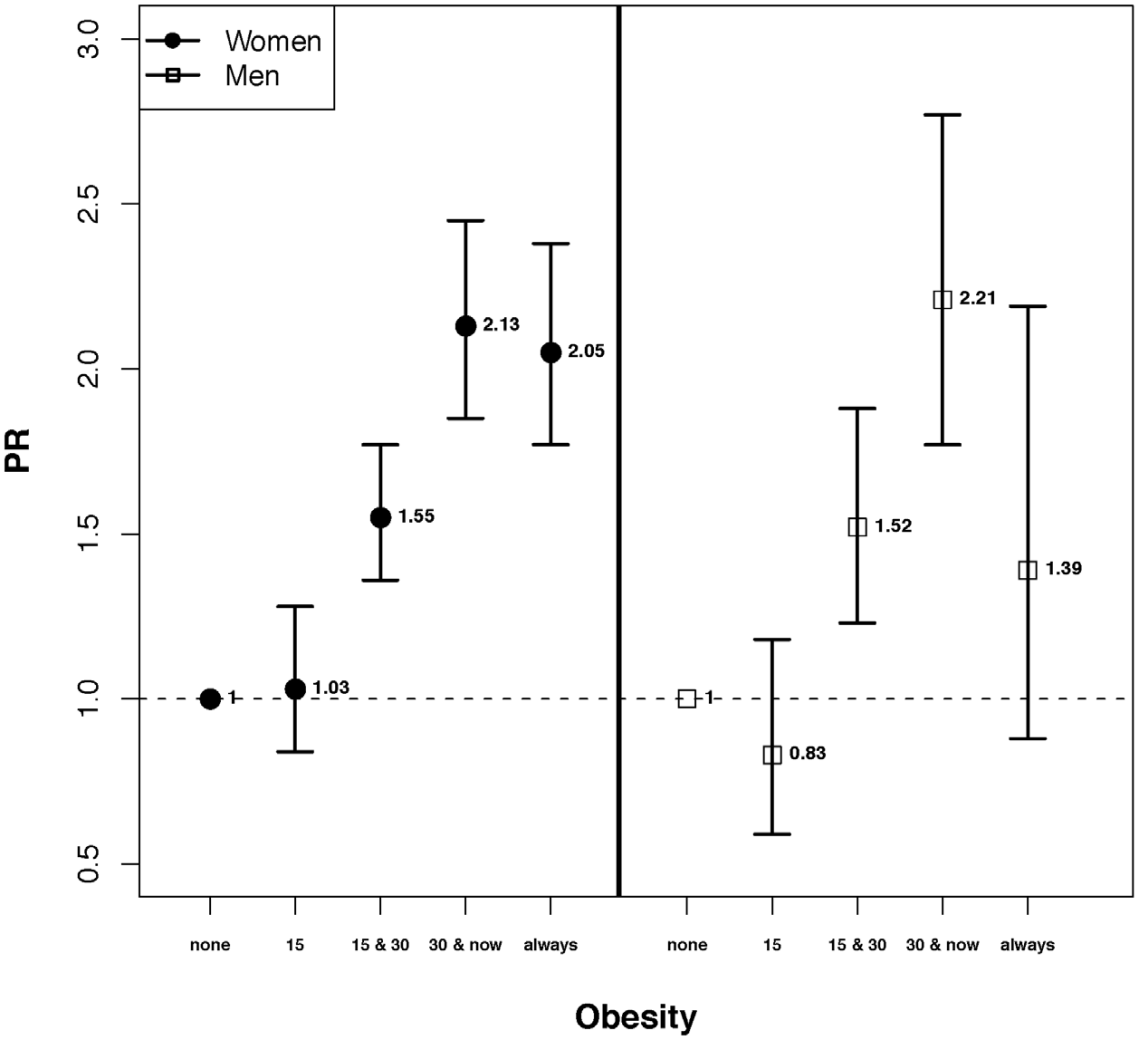
The adjusted prevalence ratios (PR) and 95% confidence intervals for diabetes in those reporting being obese at 15, 30 and cohort baseline after adjustment for age, place of residence, race, education, wealth score, physical activity, hypertension, opium, tobacco smoking, green tea consumption, black tea consumption, and DMFT score in the Golestan Cohort Study.

The cumulative effect of obesity throughout life was different between men and women. While the strength of associations were comparable for those who reported being obese at 15 and 30 or at 30 and now in both men and women, being obese throughout life (at 15, 30 and baseline) was associated with increased DM prevalence only in women (PR: 2.1; 95% CI: 1.8, 2.4) (Figure 2).

## Discussion

In this large population-based study, the overall prevalence of self-reported diabetes was 6.9%. Some of the most interesting independent correlates of DM found in this study were ethnicity, education, wealth score, opium use, consumption of green tea, body size perception at ages 15 and 30, and change in body size between 15 and 30 years.

The prevalence of DM in Iran in 2007 was estimated to be 8.7% (95% CI: 7.4%, 10.2%) according to a health survey of 4233 nationally representative Iranians [23]. However, national estimates in other studies varied between 6.1% and 9.8% [5], [8], [24]–[27]. The prevalence of self-reported diabetes age-standardized to the national population was 5.7% in our study, which is significantly less than both the national estimates and the estimated prevalence of confirmed DM in the subcohort. One of the reasons for this low prevalence of self-reported DM is the people's lack of awareness of their condition. It has been reported that one-third to one-half of diabetes cases are undiagnosed in Iran, so using a self-reported indicator could be expected to underestimate diabetes prevalence [23], [28]. In this study, only 61.5% of diabetics were aware of their disease.

The rate of self-reported diabetes was significantly less in Turkmens compared to non-Turkmens (5.9% versus 9.7%) and such differences persisted even after adjustment for potential confounders. The predicted prevalence of diabetes in Turkmenistan, north of Golestan, where 77% of the population are Turkmens, was 6.6% in 2010 [5]. Although this latter estimate may not be accurate, the similarity between our Golestan Turkmen rate and the Turkmenistan estimate is consistent with lower rates of diabetes in this ethnic group. Cultural and ethnic differences in the perception of illness and in medical-seeking behavior can be other reasons for these apparently lower prevalence rates.

As indicators of SES, both educational level and wealth score were inversely associated with DM prevalence. SES inequalities have been consistently reported to be associated with DM prevalence [25], [29], [30]. The association between SES and DM prevalence is rather complex. While some argue that secondary disability due to DM complications can lead to diminished ability to work and less educational opportunities, others attribute this finding to lower understanding of the disease status, less access to health care, being more engaged in unhealthy behaviors, and overall having a more stressful lifestyle in people with low SES [30]–[32].

Although diabetics have reported less opium use, the prevalence of self-reported diabetes increased by approximately 1.4 fold in those using opium compared to non-users. The change in the direction of the effect of opium was seen when sex, smoking status, BMI and hypertension were added into the final model. To our knowledge, this is the first report of a positive association between opium use and diabetes in a large population-based study. Although, the possibilities of reverse causation and residual confounding cannot be ruled out, it has been shown that opiates can induce insulin resistance, metabolic syndrome and diabetes [33]. The inverse association seen between smoking and diabetes prevalence can also be due to reverse causation, and the fact that sick people tend to seek more medical advice and pursue healthier lifestyle habits and thus stop smoking.

Several studies have reported a protective effect for tea consumption on incident diabetes, and the results of a recent meta-analysis indicated that drinking more than 3–4 cups of tea (black, green or oolong) per day decreases the risk of DM by 20% [34]. Despite very high intake of black tea, we did not observe any significant association for black tea consumption, but we found a positive association between green tea drinking and diabetes prevalence. Several animal and human studies have shown an antidiabetic effect for green tea polyphenols specifically epigallocatechin gallate (EGCG) [35]–[38]. EGCG induces its antidiabetic effects mostly through reduced hepatic glucose production and enhanced pancreatic function [37]. Green tea has been shown to improve glucose tolerance and has been suggested as a prophylactic agent against diabetes [35]. Our finding can again be due to reverse causation. Green tea is regarded as an herbal medicine effective for the treatment of a wide range of disease. Thus, diabetics may drink more green tea after their disease has been diagnosed, due to the common belief in the glucose-lowering effect of green tea.

Like other reports, we saw a positive association between overall and abdominal obesity and diabetes prevalence [7], [8], [28], [39]. WC has been shown to have a high predictive accuracy for diabetes detection [40], but the predictive accuracy of WC categories based on the ATP III cut-offs is questionable in developing countries [41], [42]. Several studies have reported lower cut-off points for “risky” WC in developing countries, and results of a population-based cross-sectional study of 10522 Iranian adults from Tehran suggested that compared with the ATP III criteria, optimal cut-off values should be higher in women and lower in men [43]. To overcome this issue, we looked at the quintiles of WC as well, and observed a significantly higher prevalence of diabetes in those in the highest quintile of WC compared to those in the lowest quintile in both women and men.

In addition to the commonly used measures of obesity, we were able to investigate the relationship between diabetes and previously validated pictogram estimates of body size at ages 15 and 30. The significant association seen for the previous 2 measures of obesity and DM was also seen for the pictogram estimate of body size at the age of 30. The association between pictogram category at the age of 15 and diabetes was different; the prevalence of self-reported diabetes increased in those with both extremes of body size (very lean, and very obese) at the age of 15. Although information about body size perception at all previous ages is subject to inaccurate recall, this inverse finding is not surprising, especially in the developing world. Intra-uterine growth retardation and subsequent low-birth weight have been shown to be associated with rapid weight gain, insulin resistance, further metabolic disturbances, and obesity later in life [42], [44]–[50]. Our pictogram findings provide some additional evidence for an inverse association between childhood body size and later obesity, but they need to be verified by more accurate measurements of childhood weight. Similarly, a French cohort study of around 100000 women born between 1925 and 1950, who had potentially suffered from food deprivation during World War II, showed an inverse association between menarche and early adulthood (between 20 and 25) pictogram scores and incident DM [51]. In contrast, the Nurses' Health Study II of 100000 women indicated a positive association between pictograms at ages 5 and 10 and incident type 2 DM [52]. Women in the Nurses' Health Study II were far less likely to have suffered from nutritional deficiencies during childhood.

This study has several limitations. First, the cross-sectional nature of the study makes us unable to establish any temporal relationships. For example, the associations between smoking, green tea and DM were some of the findings that can be well explained by reverse causation. Second, despite the notable agreement between self-report and medical evidence of diabetes, the self-reported nature of diabetes assessment could have induced non-differential misclassification leading to estimates biased towards the null [22], [53]. Since most associations observed in this study were significant, this potential bias means that some of the effects might actually be stronger than what we have reported. Finally, recall bias, inherent in cross-sectional studies, may explain some of the associations observed. Diabetics may have a different recall of their childhood body size compared to non-diabetics. However, it has been shown that current body size doesn't affect the accuracy of the recall childhood body size [54]. As a result, any misclassification of childhood obesity would be predominantly non-differential, and thus, would lead to attenuating the effects of producing spurious associations.

Some of the advantages of this study are its large sample size, the opportunity to examine the associations between diabetes and opium use, black and green tea consumption, information on body size perception at younger ages, and the ability to assess the link between these and diabetes. The use of PRs instead of odds ratios (ORs) was another advantage of this study. PRs are easier to interpret, and, unlike ORs, are not biased away from the null [22], [55].

In conclusion, we observed low diabetes awareness in this mainly rural population in Iran. As expected, obesity at all ages was associated with increased diabetes prevalence, but interestingly extreme leanness in childhood also showed a similar association. These findings, together with other diabetes correlates in this population such as low SES and lack of education, show the importance of improving general living conditions in diabetes prevention. Decrease in body size from childhood to early adulthood was associated with lower prevalence of diabetes, suggesting a potential role for early lifestyle modification in preventing DM.
